# Neuropharmacological effects of *Gastrodia elata* Blume and its active ingredients

**DOI:** 10.3389/fneur.2025.1574277

**Published:** 2025-04-30

**Authors:** Dong Wang, Wei Liu, MeiJuan Lu, Qiang Xu

**Affiliations:** ^1^Department of Cardiology, Second Affiliated Hospital of Tianjin University of Traditional Chinese Medicine, Tianjin, China; ^2^Tianjin University of Traditional Chinese Medicine, Tianjin, China

**Keywords:** *Gastrodia elata* Blume, neurological diseases, pharmacological mechanism, Neuropharmacological, active ingredients

## Abstract

*Gastrodia elata* Blume (GE), a traditional Chinese medicine clinically employed to treat neurological disorders, demonstrates therapeutic efficacy supported by robust clinical evidence. Nowadays, conventional pharmacotherapies for neurological conditions—such as cholinesterase inhibitors for Alzheimer’s or Ldopa for Parkinson’s—often provide limited symptom relief, exhibit side effects, and fail to halt disease w, underscoring the need for alternative strategies. The primary bioactive compounds of *Gastrodia elata* Blume (GE) include gastrodin, p-hydroxybenzyl alcohol, Vanillyl alcohol, Polysaccharides, and β-sitosterol. Modern research has demonstrated that GE and its active components exhibit neuropharmacological effects, including neuron protection, reduction of neurotoxicity, and promotion of nerve regeneration and survival. For example, Gastrodin, exerts neuroprotection by scavenging reactive oxygen species, suppressing pro-inflammatory cytokines, and enhancing GABAergic transmission, thereby alleviating oxidative stress and neuronal apoptosis. Vanillin, potentiates GABA receptor activity, enhancing inhibitory neurotransmission and reducing seizure susceptibility.GE polysaccharides modulate the gut-brain axis and suppress microglial activation, mitigating neuroinflammation. Current studies primarily focus on GE and its active ingredients for the treatment of neurological diseases such as Parkinson’s disease, Alzheimer’s disease, epilepsy, convulsions, depression, schizophrenia, as well as enhancing learning and memory, and preventing or treating cerebral ischemic injury. This review explores the neuropharmacological effects of GE and its active compounds, elucidates the underlying mechanisms, and suggests potential preventive and therapeutic strategies for neurological diseases using herbal remedies.

## Highlights

- Gastrodin significantly protected astrocytes exposed by regulating autophagy and apoptosis.
- Gastrodin promoted neuro-regenerative signaling cascades by controlling chaperone/proteasomal degradation pathways, inhibiting stress-related proteins, and modulating other neuroprotective genes and proteins with various regenerative modalities as well as capacities related to neuro-synaptic plasticity.
- Gastrodin extracts and its major bioactive components protect DA neurons, regulate the level of monoamines in the brain.
- P-hydroxybenzyl alcohol can penetrate the blood-brain barrier, protect against brain I/R injury and antioxidant stress and reduce inflammatory nerve injury.

## 1 Introduction

*Gastrodia elata* Blume (GE), a member of the Orchidaceae family, grows in Chinese woodlands and has long been used as a traditional herbal medicine for neurological disorders. The bioactive compounds in GE, including gastrodin, 4-hydroxybenzyl alcohol, benzyl alcohol, 4-(4-hydroxy-3-methoxybenzyl) alcohol, bis-(4-hydroxyphenyl) methane, and gastrodin, are able to cross the blood–brain barrier (BBB) (1–3). Other compounds (4-(4′-hydroxybenzyl) phenyl glucoside (gastrodin B, 1) and 1′-hydroxymethyl-phenyl 4-hydroxy-3-(4″-hydroxybenzyl) benzyl ether (gastrol B, 2)) isolated from the rhizomes of *Gastrodia elata* show strong neuroprotective effects against H₂O₂-induced damage in PC12 cells (4). Additionally, *Gastrodia elata* has been shown to modulate brain protein metabolism at the proteomic level (5). *Gastrodia elata* playing a neuropharmacological role through modulating numerous signaling pathways, like nuclear factor-erythroid 2-related factor (Nrf2), Wnt, neuronal nitric oxide synthase (nNOS), nuclear factor kappa-B (NF-κB), mitogen-activated protein kinase (MAPK) (6). Together, these findings suggest that GE is a promising candidate for treating neurological diseases.

The primary bioactive compounds of *Gastrodia elata* Blume (GE), including gastrodin, p-hydroxybenzyl alcohol (p-HB), vanillyl alcohol, polysaccharides, and β-sitosterol, collectively contribute to its neuropharmacological effects through multifaceted mechanisms. Gastrodin, the major glycoside, exhibits antioxidant and anti-inflammatory properties by modulating Nrf2/HO-1 and NF-κB pathways, reducing oxidative stress and neuroinflammation in neurodegenerative and ischemic conditions. p-HB and vanillyl alcohol, phenolic derivatives, enhance GABAergic transmission and scavenge free radicals, supporting neuroprotection and alleviating seizures or anxiety-related behaviors. Polysaccharides mitigate neuroinflammation by inhibiting microglial activation and cytokine release (e.g., TNF-α, IL-6), while also promoting synaptic plasticity via BDNF upregulation. β-sitosterol, a phytosterol, modulates cholesterol metabolism and neuronal membrane stability, synergizing with other compounds to attenuate apoptosis and mitochondrial dysfunction. The specific mechanisms by which they exert their neuropharmacological effects are shown as Table 1.

**Table 1:** The neuropharmacological mechanisms of action exerted by the primary bioactive components in GE.

Compound	Class	Neuropharmacological effects	Mechanisms
Gastrodin	Phenolic glycoside	Antioxidant, anti-inflammatory, anti-apoptotic, neuroprotection	Scavenges ROS Inhibits NF-κB/NLRP3 pathways Modulates BDNF/TrkB signaling Enhances GABAergic transmission
p-hydroxybenzyl alcohol	Phenolic compound	Neuroprotection, anti-epileptic, anti-inflammatory	Reduces glutamate excitotoxicity Suppresses TNF-α/IL-6 Inhibits mitochondrial apoptosis (↑Bcl-2, ↓Bax/caspase-3)
Vanillyl alcohol	Phenolic derivative	Anti-seizure, neuroprotection	Potentiates GABA_A_ receptors Attenuates oxidative stress
Polysaccharides	Carbohydrates	Immunomodulation, anti-neuroinflammatory	Inhibits microglial activation Reduces COX-2/iNOS expression Modulates gut-brain axis
β-sitosterol	Phytosterol	Neuroprotection, anti-inflammatory	Lowers cholesterol-induced neurotoxicity Inhibits NF-κB signaling

**Figure 1:**
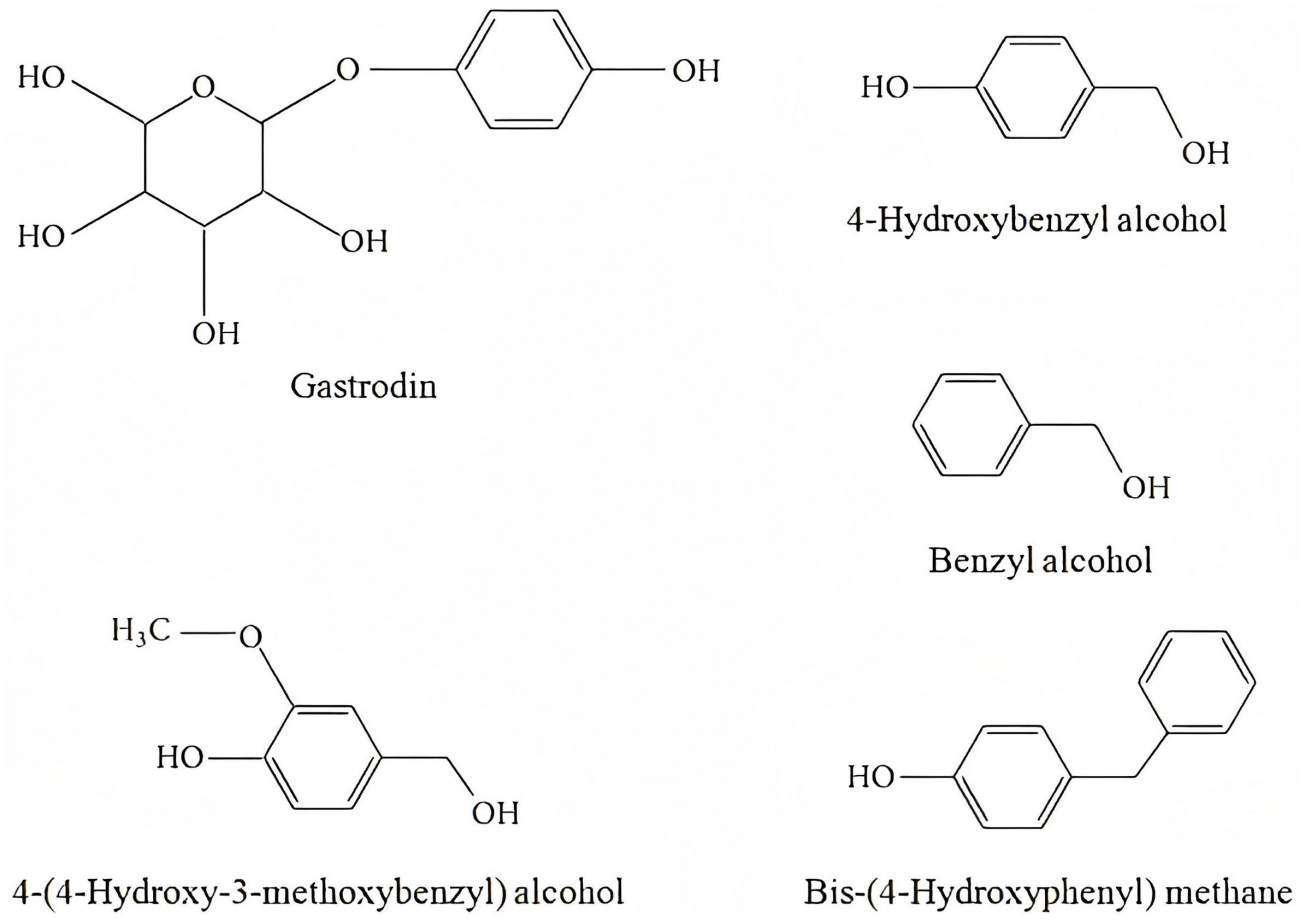
Chemical structure formula of active ingredients of GE.

Research on GE and its active compounds primarily focuses on their effects in neurological conditions, including Parkinson’s disease, Alzheimer’s disease, epilepsy and convulsions, depression, schizophrenia, cognitive dysfunction, and cerebral ischemic injury. Therefore, this review addresses each of these areas in detail, summarizing recent studies on the pharmacological effects of GE. By understanding the mechanisms of GE, we may uncover novel therapeutic opportunities for patients with neurological disorders (Figure 1).

## 2 Pharmacological mechanisms of GE and its active ingredients

### 2.1 GE and its active ingredients prevented neuronal death

Glutamate (Glu), the major excitatory neurotransmitter in the central nervous system (CNS), regulates fast synaptic transmission, neuronal plasticity, outgrowth, survival, memory, learning, and behavior, while excessive Glu triggers receptor-mediated Ca^2+^ influx through ionic channels, leading to excitotoxicity and subsequent neuronal dysfunction, damage, or death (7). As the common pathway in neurologic disorders, Glu-mediated neurotoxicity contributes to the pathogenesis of multiple neuropathological conditions (8). Glutamate (GLU)-induced neuronal death serves as a well-established injury model. In HT22 hippocampal cells, GLU exposure significantly increases both phosphorylated p38 and dephosphorylated phosphatidylinositol-3-kinase (PI3K) expression. However, pretreatment with the methanol extract of GE (MEGE) inhibits the expression of both phosphorylated p38 and dephosphorylated PI3K, thereby reducing GLU-induced HT22 hippocampal cell death. Additionally, MEGE pretreatment lowers reactive oxygen species (ROS) levels induced by GLU and enhances the expression of phosphorylated PI3K, cAMP response element-binding protein (CREB), and mature brain-derived neurotrophic factor (BDNF). These findings indicate that MEGE protects neurons primarily by upregulating the PI3K signaling pathway in conjunction with BDNF (9).GLU exposure also triggers a gradual and sustained rise in intracellular Ca^2+^ concentration, a key mechanism driving neuronal apoptosis. In IMR32 human neuroblastoma cells, the active GE components vanillin (VAN) and p-hydroxybenzaldehyde (p-HB) significantly inhibit both GLU-induced intracellular Ca^2+^ elevation and neuronal apoptosis (10). Furthermore, in PC12 cells subjected to serum deprivation, GE targets the adenosine A (2A) receptor (A (2A)-R), promoting cAMP formation, increasing protein kinase A (PKA) activity, and enhancing CREB phosphorylation, which collectively inhibit apoptosis in PC12 cells (11).

In PC12 cells, gastrodin, a key active compound in GE, effectively inhibits GLU-induced oxidative stress. Specifically, gastrodin reduces GLU-induced intracellular Ca^2+^ influx, thereby blocking the activation of calmodulin-dependent kinase II (CaMKII) and apoptosis signaling-regulating kinase-1 (ASK-1). Additionally, gastrodin suppresses the phosphorylation of p38 mitogen-activated protein kinase (MAPK), p53, caspase-3, and cytochrome C, while decreasing the GLU-induced bax/bcl-2 ratio in PC12 cells (12). Another study demonstrated that GE polysaccharides protect PC12 cells from corticosterone-induced apoptosis by inhibiting the endoplasmic reticulum (ER) stress-mediated pathway (13).

Autophagy, a programmed cell death mechanism, is also influenced by GE. Gastrodin significantly protects astrocytes from autophagy and apoptosis when exposed to lipopolysaccharides (LPS). Further analysis shows that gastrodin reduces the expression of LC3-II, P62, and Beclin-1, protecting astrocytes from autophagy. Gastrodin also modulates the Bcl-2 and Bax signaling pathways to prevent astrocyte apoptosis (14). Network pharmacology studies (15) indicate that alexandrin (an active GE component) enhances STAT3 expression to exert anti-inflammatory and anti-apoptotic effects, while para-hydroxybenzaldehyde and gastrodin inhibit myeloperoxidase (MPO) and matrix metalloproteinase-9 (MMP9) expression, respectively, attenuating neuroinflammation and blood–brain barrier disruption (15). These actions help protect ischemic neurons, contributing to the anti-cerebral ischemia/reperfusion injury (CIRI) effects of GE (15).

### 2.2 GE and its active ingredients reduced neurotoxicity

Several studies have demonstrated that gastrodin can mitigate amyloid β (Aβ) (1–42)-induced neurotoxicity in primary neural progenitor cells (NPCs). Gastrodin enhances cell viability, reduces the release of pro-inflammatory cytokines and nitric oxide (NO), and alleviates Aβ (1–42)-induced apoptosis in NPCs. One study found that GAS suppressed NLRP3 inflammasome signaling pathway, and therefore suppressed pyroptosis and exerted neuroprotective effect (16). Additionally, gastrodin reverses the Aβ (1–42)-induced increase in phosphorylation of MEK-1/2, extracellular signal-regulated kinases (ERK), and c-Jun N-terminal kinase (JNK). Apoptosis plays a critical role in ischemia/reperfusion (I/R)-induced neuronal death. In a middle cerebral artery occlusion (MCAO) rat model, gastrodin preserved the expression of the anti-apoptotic protein Bcl-2 while suppressing the expression of the pro-apoptotic Bax protein. It also reduced the levels of cleaved caspase-3, a key marker of apoptosis, induced by cerebral I/R (17). In Aβ (1–42)-injected C57BL/6 mice, gastrodin promoted hippocampal neurogenesis (18). Yang et al. (19) demonstrates that gastrodin mitigates methamphetamine-induced autophagic neurotoxicity in SH-SY5Y dopaminergic neurons by dose-and time-dependently suppressing LC3B/Beclin-1 overexpression and autophagosome formation, mechanistically linked to AKT/mTOR pathway activation. Additionally, gastrodin effectively counteracted neurotoxicity induced by hypoxia, glutamate, and N-methyl-D-aspartate (NMDA) receptors in primary rat cortical neurons (20, 123). Under ischemic conditions, inducible nitric oxide synthase (iNOS) astrocytes typically exhibit increased expression of, leading to excessive NO production, which contributes to neurotoxicity. Gastrodin protects astrocytes from I/R injury by inhibiting iNOS expression and reducing NO-induced neurotoxicity (21). Additionally, gastrodin can downregulate NLRP3, NLRC4, caspase-1, and IL-18 in astrocytes subjected to ischemic stress, while also reducing STAT3 and NF-κB pathway activity. Furthermore, gastrodin regulates the PI3K/AKT-Sirt3 axis, enhancing antioxidant defenses by suppressing ROS production and promoting FOXO3a phosphorylation in activated microglia, thereby alleviating oxidative stress and inflammation.

Lead is a well-known environmental toxin that causes significant damage to the nervous system. Exposure to lead impairs synaptic plasticity in the hippocampal CA1 region of rats at postnatal day 22 (P22), but gastrodin effectively mitigates these lead-induced impairments. The study showed that lead exposure disrupts synaptic plasticity, reduces BDNF levels, and triggers neuroinflammation, apoptotic neurodegeneration, and deficits in neuronal plasticity, cognition, and brain development (22). Gastrodin reduces the accumulation of phosphorylated tau (p-tau) and amyloid-beta (Aβ), inhibits lead-induced brain inflammation, and increases the expression of NR2A and BDNF. Additionally, gastrodin alleviates oxidative stress via nuclear factor erythroid 2-related factor 2 (Nrf2)-mediated antioxidant signaling modulation, activates the Wnt/β-catenin pathway, and decreases the expression of the Wnt inhibitor Dickkopf-1 (Dkk-1) (23). These findings suggest that gastrodin may offer therapeutic potential for lead-induced neurotoxicity (23, 24). Several phenolic compounds isolated from GE have also been shown to counteract KCl-induced neurotoxicity in PC12 cells (25). Furthermore, GE effectively reduce (MPP+)-induced cytotoxicity in human dopaminergic SH-SY5Y cells (26). Both gastrodin and vanillyl alcohol protect against MPP(+)-induced cytotoxicity by upregulating the Bcl-2 protein, thereby inhibiting the apoptotic pathway in Parkinson’s disease cell models (27–29).

### 2.3 GE and its active ingredients promote nerve regeneration and survival

Research indicates that GE stimulates the proliferation and differentiation of human neural stem cells (NSCs) derived from embryonic stem cells (30).

Using an iTRAQ (isobaric tag for relative and absolute quantitation)-based proteomics approach, researchers identified 406 proteins modified by GE treatment in differentiated human neuronal SH-SY5Y cells. These findings suggest that GE promotes neuroregenerative signaling pathways by regulating chaperone/proteasomal degradation, inhibiting stress-related proteins, and modulating other neuroprotective genes that enhance neuroplasticity and regeneration (31, 32).GE enhances neurogenesis by activating pathways like BDNF/TrkB and Wnt/β-catenin, promoting neural stem/progenitor cell proliferation in hippocampal and subventricular zones. Additionally, gastrodin (GAS) and 4-hydroxybenzyl alcohol (HBA), active compounds in GE, improve learning and memory, reduce neuronal damage and Aβ deposition, and decrease Tau phosphorylation. In rats, these compounds also improve energy metabolism in the brain and protect cells from mitochondrial dysfunction caused by H₂O₂-induced oxidative stress (33). Studies further indicate (34) that GE exerts neuroprotective effects through upregulation of Ncam1, Hsp90aa1, Tpi1, and Ppia alongside downregulation of Sept2 and Uchl1, restoring metabolic balance and promoting neuronal survival (34).

**Figure 2:**
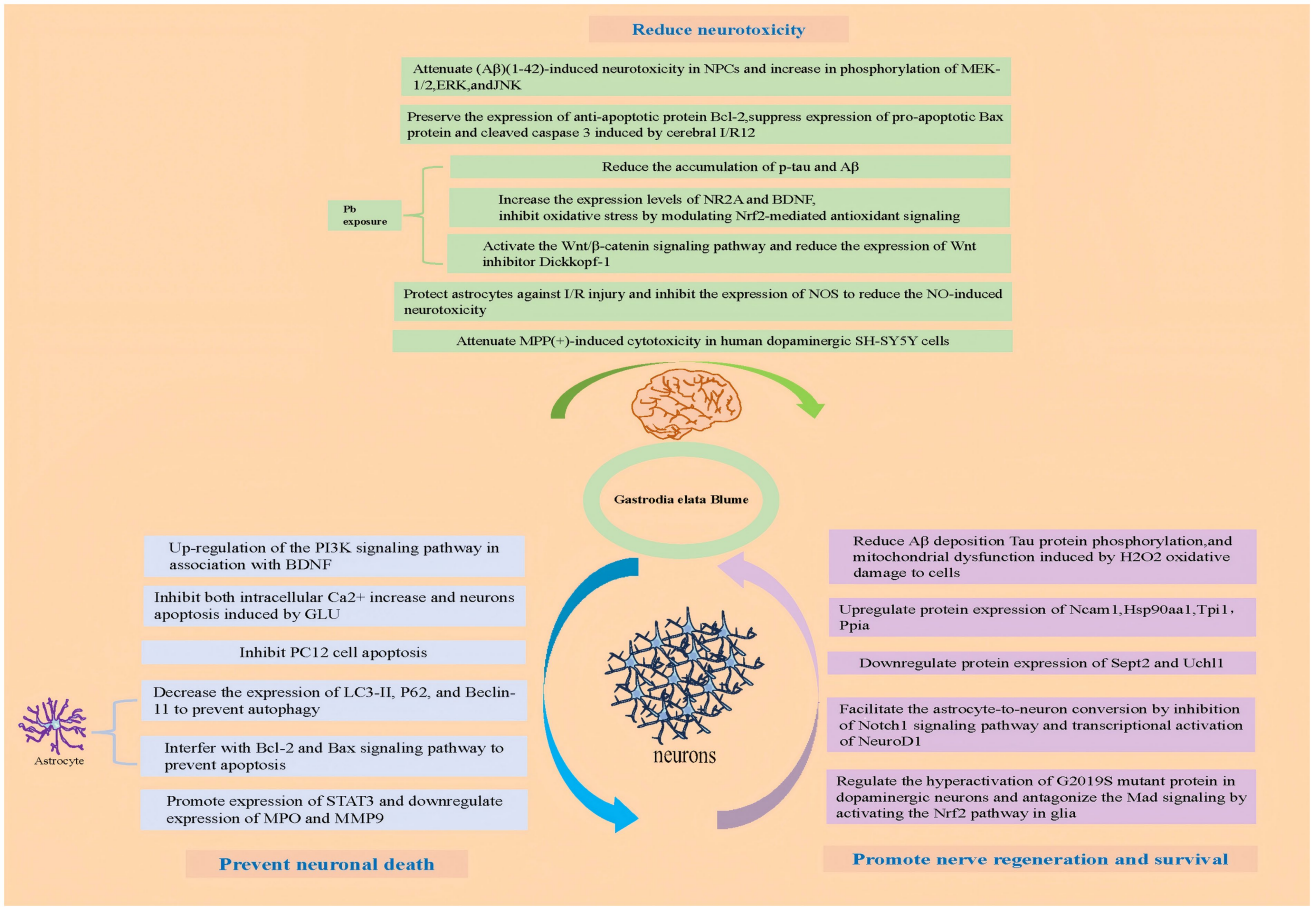
Pharmacological mechanisms of GE and its active ingredients.

In another study, p-HBA promoted astrocyte-to-neuron conversion by inhibiting the Notch1 signaling pathway and activating NeuroD1 transcription. Within 14 days, these converted neurons matured, demonstrating GE’s potential for neuronal differentiation (35). Moreover, GE has been found to regulate the hyperactivation of G2019S, a mutant protein in dopaminergic neurons, and to counteract Mad signaling via Nrf2 pathway activation in glial cells, both of which contribute to its neuroprotective effects (36). These effects hold significant implications for regenerative medicine, particularly in treating neurodegenerative diseases (e.g., Alzheimer’s, Parkinson’s) and brain injuries, as GE may stimulate endogenous repair mechanisms or synergize with stem cell therapies to restore neural function. Further exploration of GE-derived compounds could yield novel neuroregenerative therapeutics (Figure 2).

## 3 GE and its active ingredients in treating neurological diseases

### 3.1 Anti-Parkinson’s disease

Neuroinflammation in PD is mostly linked to the reactive state of glial cells in the brain. The secretion of pro-inflammatory cytokines such as Interleukin-1β (IL-1β), IL-6, and tumor necrosis factor-α (TNF-α) to the brain microenvironment may accelerate neurodegeneration in PD (37). Reducing microglia-mediated neuroinflammation can thus decrease the degeneration of dopaminergic neurons (38). A Parkinson’s disease (PD) cell model was established by exposing PC12 cells to rotenone. In this model, neuroinflammation was characterized by elevated levels of pro-inflammatory cytokines, including IL-1β, IL-6, and TNF-α, alongside reduced resting microglia in the substantia nigra (SN) of rotenone-induced PD rats (39). Gastrodin treatment effectively reduced these inflammatory markers. Furthermore, a bibenzyl compound isolated from GE dose-dependently mitigated rotenone-induced apoptosis and oxidative stress in PC12 cells (40). Another widely used *in vitro* PD model involves 1-methyl-4-phenylpyridinium (MPP+)-treated MN9D dopaminergic cells. In this model, GE extracts and their primary bioactive components protected dopaminergic MN9D cells from MPP + -induced apoptosis by alleviating oxidative stress and modulating the apoptotic pathways (41). A separate study showed that gastrodin (GTD) alleviated PD-related motor deficits and dopaminergic neuronal damage by enhancing MEK-dependent regulation of VMAT2, which is involved in dopamine homeostasis (42). Additionally, in a rotenone-induced PD rat model, co-treatment with vanillin and levodopa-carbidopa significantly improved motor deficits and reduced oxidative stress markers, such as lipid peroxidation, and increased levels of GSH and catalase in the brain (43).

The underlying mechanism of GE’s neuroprotective effects may involve multiple pathways, including the enhancement of the body’s antioxidant capacity, protection of dopaminergic (DA) neurons, regulation of brain monoamine levels, inhibition of various apoptosis-related signaling pathways, and activation of Wnt signaling pathways (44, 45). Additionally, GE may regulate the Keap1-Nrf2/HO-1 pathway, leading to increased expression of downstream antioxidant genes and enhanced superoxide dismutase (SOD) enzyme activity (46). In a 6-OHDA-induced PD rat model, vanillin treatment significantly reduce apomorphine-induced contralateral rotation and maintained dopamine levels (47).

Moreover, gastrodin increases the expression of HO-1 through the activation of the p38 MAPK/Nrf2 signaling pathway, which protects SH-SY5Y cells from MPP + -induced oxidative stress and cell death in a PD cell model (28). In the subchronic MPTP-induced mouse PD model, gastrodin also exhibited neuroprotective effects, improving bradykinesia and motor impairments (27).

Currently, L-3,4-dihydroxyphenylalanine (L-DOPA), a dopamine precursor, is the gold-standard treatment for PD. However, long-term use of L-DOPA can lead to L-DOPA-induced dyskinesia (LID). Therefore, it is crucial to identify safe and effective alternative treatments. Some studies suggest that GE may benefit PD patients by modulating the insulin-like DAF-2/DAF-16 signaling pathway (48, 49).

### 3.2 Anti-Alzheimer’s disease

As previously described, amyloid β (Aβ)-induced neurotoxicity plays a central role in the pathogenesis of Alzheimer’s disease (AD). Several studies have demonstrated that GE significantly reduces Aβ-induced neuronal cell death *in vitro* (50, 51). One study showed that 4,4′-methylenediphenol, a key active component of *Gastrodia elata*, enhances the expression of DAF-16, SOD-3, SKN-1, and GST-4 by activating the DAF-16/FOXO and SKN-1/NRF2 signaling pathways. These actions improve antioxidant capacity, which, in turn, reduces ROS and Aβ aggregation, thereby alleviating Aβ toxicity (52). Further investigations (53) suggested that the inhibitory mechanisms of GE may involve the reduction of β-and γ-secretase activities. Zhang et al. (54) reported that gastrodin, another active compound in *Gastrodia elata*, suppresses β-secretase expression by inhibiting the protein kinase/eukaryotic initiation factor-2α (PKR/eIF2α) pathway in an AD mouse model. In the Tg2576 mouse model of AD, gastrodin significantly improved memory impairments, as assessed by the Morris water maze and probe tests. Additionally, gastrodin enhanced cell viability in an Aβ25-35-induced cell culture model of AD, reducing lactate dehydrogenase (LDH) release and thereby protecting neurons from Aβ toxicity (55). Moreover, gastrodin significantly reduced Aβ deposition and glial activation in the brains of these mice (56). Further research indicated that gastrodin alleviates intracellular oxidative stress in the hippocampi of Tg2576 mice and mitigates memory deficits by inhibiting the PKR/eIF2α pathway (54).

We established a rat model of AD by injecting Aβ25-35 into the bilateral hippocampi. The rats were then intragastrically administered GE, and the results demonstrated that GE treatment significantly improved spatial memory. Moreover, GE treatment markedly reduced amyloid deposits in the hippocampus, increased choline acetyltransferase expression in the medial septum and hippocampus, and inhibited acetylcholinesterase activity in the prefrontal cortex, medial septum, and hippocampus of these AD rats (57). *Gastrodia elata* also alleviates cognitive deficits in vascular dementia (VD) rats by reducing the accumulation of toxic substances, including Aβ and tau proteins (58), and by decreasing excessive autophagy and neuronal cell apoptosis (59). Another study found that HBA effectively increased neurotrophic factors while reducing inflammatory markers, thus improving both working and spatial memory in AD model mice (60).

### 3.3 Anti-epilepsy and anti-convulsions

Several studies have established that GE and its components exhibit anti-epileptic and anti-convulsive properties in *in vivo* models. A clinical study has also demonstrated that vanillin, a key component of GE, has anti-epileptic effects (61). Furthermore, GE has been shown to scavenge ROS and reactive nitrogen species (RNS) and to prevent the occurrence of epileptic discharges in iron-injected rat models (62). Other studies suggest that GE may modulate GABA levels, which could contribute to its anti-epileptic effects (63–65). *In vivo*, gastrodin has been shown to inhibit the activities of enzymes responsible for GABA degradation—namely, GABA transaminase (GABA-T), succinic semialdehyde reductase (SSAR), and SSADH—in the hippocampus of seizure-sensitive gerbils (63). Yang et al. (66) demonstrates that gastrodin ameliorates lithium-pilocarpine-induced seizure severity, and exerts neuroprotective effects against hippocampal neuronal damage at acute/subacute phases, mediated through upregulation of GABAA receptor expression, highlighting its potential as a novel therapeutic agent derived from traditional Chinese medicine for epilepsy management. Additionally, brain inflammation plays a crucial role in epileptogenesis. Chen et al. (67) demonstrated that gastrodin could reduce levels of pro-inflammatory cytokines, such as interleukin-1beta (IL-1β) and tumor necrosis factor-alpha (TNF-α), while reversing the decrease in the anti-inflammatory cytokine interleukin-10 (IL-10) in the brains of PTZ-induced mice.

In an *in vivo* experiment, mice were treated with the ether fraction of methanol extracts (EFME) of GE for 14 days prior to kainic acid (KA) injection. The EFME of GE significantly delayed the onset of neurobehavioral changes and notably reduced the severity of convulsions and hippocampal neuronal damage in the CA1 and CA3 regions (68). In another study, oral administration of GE significantly reduced the frequency of wet dog shakes (WDS), paw tremors (PT), and facial myoclonia (FM) in KA-treated rats. Additionally, GE delayed the onset of WDS in these rats, further supporting its anti-convulsive effects (69). GE also modulated the expression of activator protein 1 (AP-1) through the JNK signaling pathway, which may underlie one of its anti-convulsive mechanisms (70). Furthermore, some studies suggest that the anti-convulsive effects of GE could be attributed to its vanillyl alcohol (VA) component (71, 72).

### 3.4 Anti-depression

Numerous *in vivo* studies have demonstrated that GE exhibits anti-depressant effects (73). The GE extract significantly increased DA levels while decreasing the concentration of 3,4-dihydroxyphenylacetic acid (DOPAC), leading to a reduction in DA turnover in the striatum of Sprague–Dawley rats (74). Similarly, the GE extract raised serotonin (5-HT) levels in the frontal cortex and DA levels in the striatum. It also decreased the ratios of 5-HIAA/5-HT and (DOPAC + HVA)/DA, indicating reduced turnover of both 5-HT and DA in rats during the forced-swimming test (FST) (75). Another study found that in rats exposed to the unpredictable chronic mild stress (UCMS) model, the GE extract significantly reversed sucrose preference and other abnormal behaviors. It also restored cerebral turnover rates of 5-HT and DA while lowering serum corticosterone levels (76). These findings suggest that the anti-depressant effects of GE may involve the modulation of both serotonergic and dopaminergic systems. Additionally, proteomic and bioinformatics analyses indicated that the GE extract influenced the core protein network, particularly by down-regulating the Slit-Robo pathway. Since the Slit-Robo pathway is involved in neuronal cytoskeletal remodeling, these results imply that both the Slit-Robo pathway and neuronal cytoskeletal remodeling may contribute to the anti-depressant-like effects of the GE extract (77, 78).

Network pharmacology predictions suggest that *G. elata* exerts its anti-depressant effects through reticulon 4 receptors (RTN4R) and apoptosis-related targets (79). Parishin C (Par), a prominent bioactive compound in *G. elata*, has been shown to significantly alleviate depression-like behaviors induced by chronic social defeat stress (CSDS) in mice. This effect was accompanied by a reduction in serum corticosterone levels and an increase in the concentrations of serotonin (5-HT), DA, and norepinephrine (NE) in the hippocampus and prefrontal cortex (80).GE also engages CB1R-dependent PKA/RhoA signaling to restore synaptic protein expression and dendritic spine density in hippocampal neurons, mitigating post-stroke depressive behaviors linked to neuroinflammation.

Furthermore, *G. elata* improved depression-like behaviors and reversed stress-induced elevations of corticosterone in C57BL/6 mice exposed to the CSDS model. It achieved this by increasing the protein expression of BDNF and enhancing the phosphorylation ratio of cAMP CREB and protein kinase B (Akt) in the hippocampus (81). In a chronic unpredictable stress (CUS)-induced depression rat model, the expression of glial fibrillary acidic protein (GFAP) and BDNF was reduced in the hippocampus; however, gastrodin reversed these changes. *In vitro*, gastrodin also improved levels of phospho-ERK1/2 and BDNF in hippocampal-derived astrocytes. These findings suggest that the anti-depressant effects of gastrodin are linked to the enhancement of BDNF levels and the modulation of astrocyte activation (21). In conclusion, The antidepressant mechanism involves an increase in the neurotransmitters, anti-inflammatory effects, increases in the number of new neurons, the rearrangement of the nerve cytoskeleton, and regulation of the expression of related inflammatory factors (82).

### 3.5 Anti-schizophrenia

The 5-HT (1A) receptors play a crucial role in the pathophysiology of schizophrenia, and since GE modulates the serotonergic system, we investigated its effects on abnormal behavior in mice induced by phencyclidine (PCP). GE significantly attenuated these abnormal behaviors, with effects comparable to those of 8-OH-DPAT, a 5-HT (1A) receptor agonist. Furthermore, the effects of GE were reversed by WAY 100635, a 5-HT (1A) receptor antagonist. These findings suggest that GE exerts an anti-schizophrenic effect through the activation of 5-HT (1A receptors) (83). Similarly, parishin C, a major component of GE, exhibits comparable pharmacological effects (84). GE also downregulates the Slit-Robo pathway, linked to neuronal cytoskeletal remodeling, and reduces stress-induced corticosterone, addressing neuroinflammation and oxidative stress.

### 3.6 Improvement in learning and memory

Several preclinical studies have demonstrated that GE and its extracts can improve learning and memory deficits in rats (59, 85–88). The phenolic compound 4-hydroxybenzyl methyl ether (HBME), isolated from GE, significantly increased step-through latency at all three stages of memory (acquisition, consolidation, and retrieval) in the step-through passive avoidance task in mice. Furthermore, the pharmacological effects of HBME were reversed by the dopamine D1 receptor antagonist SCH23390 or the PKA antagonist H-89. HBME also increased the phosphorylation of PKA and cAMP CREB in the cortex and hippocampus. Notably, these enhancing effects were blocked by SCH23390. In contrast, HBME alleviated memory impairments induced by SCH23390 (89). Gastrodin effectively mitigated 3,3′-iminodipropionitrile (IDPN)-induced working memory deficits in the Y-maze task in rats. Additionally, gastrodin prevented the reduction of DA and its metabolites, as well as the increase in the DA turnover ratio [(DOPAC + HVA)/DA], induced by IDPN. Gastrodin also preserved dopamine D2 receptor and dopamine transporter protein levels in the hippocampus of rats (90). In the context of ADHD treatment, gastrodin may enhance DA release and transport by modulating DA receptor function, while also inhibiting proinflammatory cytokines and GIRK channels (91). These findings suggest that the effects of GE and its components on cognitive function are likely mediated, at least in part, by dopaminergic neurotransmitter signaling (92).

Additionally, gastrodin inhibited the reduction of γ-aminobutyric acid (GABA) levels and the increase in α2 GABAA receptor protein expression in the prefrontal cortex and hippocampus of rats induced by IDPN (93). These findings suggest that the effects of GE and its components on cognitive function may, in part, involve the normalization of the GABAergic system (94). Furthermore, GE and its components enhanced cognitive function by increasing plasma adrenal steroid levels (95), inhibiting β-site APP-cleaving enzyme 1 activity, and promoting neuroprotective α-secretase activity (96).

### 3.7 Prevention and treatment of cerebral ischemic injury

GE and its extracts have demonstrated the ability to prevent and treat cerebral ischemic injury in numerous studies (97). Wang et al. (98) indicate that GAS significantly improves neurological function and neuronal survival in a permanent cerebral infarction model, potentially through mechanisms involving suppression of inflammatory responses, inhibition of apoptosis, and enhancement of revascularization in the ischemic hemisphere. Administration of gastrodin prior to ischemia significantly reduced glutamate elevation during the postischemic period and increased extracellular GABA levels during reperfusion in the rat hippocampus. This shift led to a decrease in the glutamate/GABA ratio during both ischemia and reperfusion (99–103). Gastrodin also markedly reduced infarct and edema volumes while improving neurological function (104). Additionally, gastrodin enhanced the secretion of brain-derived neurotrophic factor, which further contributed to the recovery of neurological function and protected neural cells from injury (105, 106). EAA-induced neurotoxicity is considered a primary pathological mechanism in ischemic brain damage. Gastrodin significantly inhibited the release of cerebral amino acids, particularly EAAs, thereby modulating the imbalance between EAAs and inhibitory amino acids (IAAs) during I/R (21).

Furthermore, 3,4-dihydroxybenzaldehyde (DBD), an active compound in GE, significantly reduced infarct volume and alleviated neurological deficits in rats. This effect was mediated by the inhibition of microglia activation, selective modulation of microglial polarization, and a reduction in inflammatory mediators and cytokine production through the suppression of MAPK and NF-κB activation (107). P-HBA, an active compound in GE, prevents cerebral ischemic injury by modulating cytoprotective genes, including Nrf2 and PDI, as well as neurotrophic factors (108). PHBA penetrates the BBB, protects against brain I/R injury, reduces oxidative stress, and mitigates inflammatory neural damage (109). Additionally, HBA and other active GE ingredients increase the expression of genes encoding antioxidant and anti-inflammatory proteins (107, 110, 111). For instance, they promote PSD-95-AMPAR activity, elevate protein expression levels of PSD-95 and GluA1, and suppress apoptosis-related pathways (112) to alleviate cerebral ischemic injury (113).

**Figure 3:**
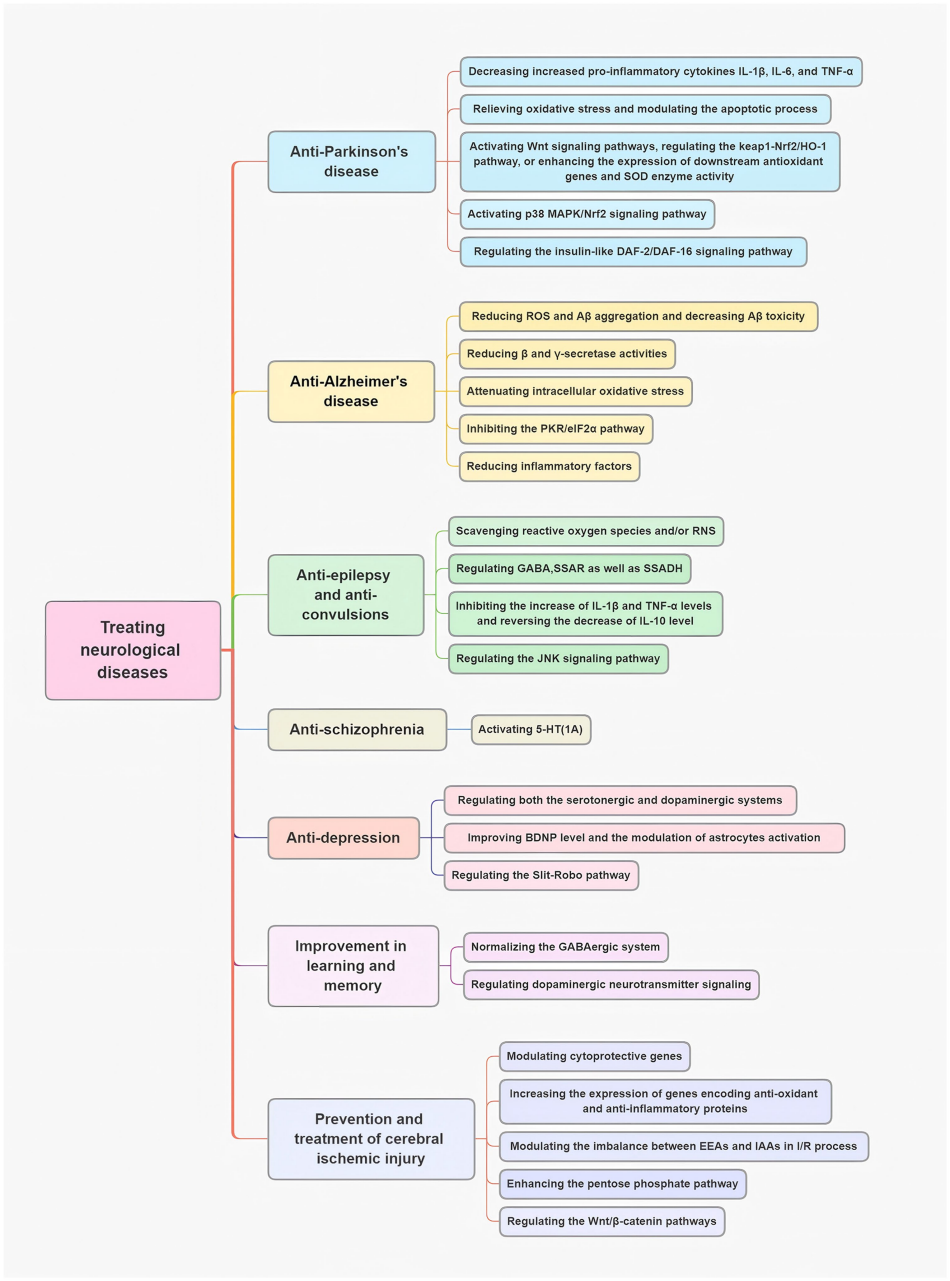
The mechanism of GE and its active ingredients in the treatment of neurological diseases.

Evidence suggests that enhancing the pentose phosphate pathway may serve as a therapeutic target for ischemic brain injury (114). Gastrodin increases ribose 5-phosphate levels, influencing the pentose phosphate pathway and improving ischemic brain damage (115). The Wnt/β-catenin signaling pathway also plays a crucial role in regulating hippocampal development and synaptogenesis (116). One study showed that gastrodin enhances neurogenesis and reduces ischemic damage in a cerebral ischemia model through activation of the Wnt/β-catenin pathway (117). Additionally, gastrodin accelerates hippocampal neurogenesis after cerebral ischemia via the PDE9-cGMP-PKG signaling pathway (118) (Figure 3).

## 4 Conclusion

GE, a traditional Chinese medicine, has been used clinically for thousands of years (119). Recent studies demonstrate that GE and its active compounds exhibit neuropharmacological effects, including neuroprotection, reduction of neurotoxicity, and promotion of nerve regeneration and survival. For currently incurable neurological disorders such as AD, PD, epilepsy, convulsions, depression, schizophrenia, and cerebral ischemic injury, available therapies offer only limited symptom relief or modest slowing of disease progression. Thus, there is an urgent need for new therapeutic agents that can effectively treat and support recovery from these neurological conditions. A series of studies confirms that GE and its active ingredients possess a range of beneficial effects, including anti-Parkinson’s, anti-Alzheimer’s, anti-epileptic, anticonvulsant, antidepressant, antipsychotic, cognitive-enhancing, and neuroprotective actions, especially against cerebral ischemic injury. As a result, GE shows promise as a potential alternative treatment for various intractable neurological diseases. Notably, gastrodin, a major active component of GE, has seen extensive clinical application, although its pharmacological properties require further exploration (120–122). In this review, we summarize the applications and mechanisms of GE and its active ingredients in neurological diseases, aiming to provide new therapeutic strategies for these challenging conditions.

## References

[1] Lin LC Chen YF Tsai TR Tsai TH. Analysis of brain distribution and biliary excretion of a nutrient supplement, gastrodin, in rat. Anal Chim Acta. (2007) 590:173–9. doi: 10.1016/j.aca.2007.03.035, PMID: 17448342

[2] Wang Q Chen G Zeng S. Distribution and metabolism of gastrodin in rat brain. J Pharm Biomed Anal. (2008) 46:399–404. doi: 10.1016/j.jpba.2007.10.017, PMID: 18053670

[3] Lin LC Chen YF Lee WC Wu YT Tsai TH. Pharmacokinetics of gastrodin and its metabolite p-hydroxybenzyl alcohol in rat blood, brain and bile by microdialysis coupled to LC-MS/MS. J Pharm Biomed Anal. (2008) 48:909–17. doi: 10.1016/j.jpba.2008.07.013, PMID: 18757149

[4] Zhang ZC Su G Li J Wu H Xie XD. Two new neuroprotective phenolic compounds from *Gastrodia elata*. J Asian Nat Prod Res. (2013) 15:619–23. doi: 10.1080/10286020.2013.791286, PMID: 23659598

[5] Manavalan A Feng L Sze SK Hu JM Heese K. New insights into the brain protein metabolism of *Gastrodia elata*-treated rats by quantitative proteomics. J Proteome. (2012) 75:2468–79. doi: 10.1016/j.jprot.2012.02.029, PMID: 22402058

[6] Gong MQ Lai FF Chen JZ Li XH Chen YJ He Y. Traditional uses, phytochemistry, pharmacology, applications, and quality control of *Gastrodia elata* Blume: a comprehensive review. J Ethnopharmacol. (2024) 319:117128. doi: 10.1016/j.jep.2023.117128, PMID: 37689324

[7] Zhang LN Hao L Wang HY Su HN Sun YJ Yang XY. Neuroprotective effect of resveratrol against glutamate-induced excitotoxicity. Adv Clin Exp Med. (2015) 24:161–5. doi: 10.17219/acem/38144, PMID: 25923101

[8] Lipton SA Rosenberg PA. Excitatory amino acids as a final common pathway for neurologic disorders. N Engl J Med. (1994) 330:613–22. doi: 10.1056/NEJM199403033300907, PMID: 7905600

[9] Han YJ Je JH Kim SH Ahn SM Kim HN Kim YR. *Gastrodia elata* shows neuroprotective effects via activation of PI3K signaling against oxidative glutamate toxicity in HT22 cells. Am J Chin Med. (2014) 42:1007–19. doi: 10.1142/S0192415X14500633, PMID: 25004888

[10] Lee YS Ha JH Yong CS Lee DU Huh K Kang YS. Inhibitory effects of constituents of *Gastrodia elata* Bl. On glutamate-induced apoptosis in IMR-32 human neuroblastoma cells. Arch Pharm Res. (1999) 22:404–9. doi: 10.1007/BF02979066, PMID: 10489882

[11] Tsai CF Huang CL Lin YL Lee YC Yang YC Huang NK. The neuroprotective effects of an extract of *Gastrodia elata*. J Ethnopharmacol. (2011) 138:119–25. doi: 10.1016/j.jep.2011.08.064, PMID: 21925258

[12] Jiang G Wu H Hu Y Li J Li Q. Gastrodin inhibits glutamate-induced apoptosis of PC12 cells via inhibition of CaMKII/ASK-1/p 38 MAPK/p53 signaling cascade. Cell Mol Neurobiol. (2014) 34:591–602. doi: 10.1007/s10571-014-0043-z, PMID: 24619207 PMC11488867

[13] Zhou B Tan J Zhang C Wu Y. Neuroprotective effect of polysaccharides from *Gastrodia elata* blume against corticosterone-induced apoptosis in PC12 cells via inhibition of the endoplasmic reticulum stress-mediated pathway. Mol Med Rep. (2018) 17:1182–90. doi: 10.3892/mmr.2017.7948, PMID: 29115511

[14] Wang XS Tian Z Zhang N Han J Guo HL Zhao MG. Protective effects of Gastrodin against autophagy-mediated astrocyte death. Phytother Res. (2016) 30:386–96. doi: 10.1002/ptr.5538, PMID: 26643508

[15] Luo Y Chen P Yang L Duan X. Network pharmacology and molecular docking analysis on molecular targets and mechanisms of *Gastrodia elata* Blume in the treatment of ischemic stroke. Exp Ther Med. (2022) 24:742. doi: 10.3892/etm.2022.11678, PMID: 36569043 PMC9764286

[16] Yang F Li G Lin B Zhang K. Gastrodin suppresses pyroptosis and exerts neuroprotective effect in traumatic brain injury model by inhibiting NLRP3 inflammasome signaling pathway. J Integr Neurosci. (2022) 21:72. doi: 10.31083/j.jin2102072, PMID: 35364660

[17] Peng Z Wang S Chen G Cai M Liu R Deng J. Gastrodin alleviates cerebral ischemic damage in mice by improving anti-oxidant and anti-inflammation activities and inhibiting apoptosis pathway. Neurochem Res. (2015) 40:661–73. doi: 10.1007/s11064-015-1513-5, PMID: 25582916

[18] Li M Qian S. Gastrodin protects neural progenitor cells against amyloid β (1-42)-induced neurotoxicity and improves hippocampal neurogenesis in amyloid β (1-42)-injected mice. J Mol Neurosci. (2016) 60:21–32. doi: 10.1007/s12031-016-0758-z, PMID: 27112440

[19] Yang G Zeng X Li J Leung CK Zhang D Hong S. Protective effect of gastrodin against methamphetamine-induced autophagy in human dopaminergic neuroblastoma SH-SY5Y cells via the AKT/mTOR signaling pathway. Neurosci Lett. (2019) 707:134287. doi: 10.1016/j.neulet.2019.134287, PMID: 31128157

[20] Wong SB Hung WC Min MY. The role of Gastrodin on hippocampal neurons after N-methyl-D-aspartate excitotoxicity and experimental temporal lobe seizures. Chin J Physiol. (2016) 59:156–64. doi: 10.4077/CJP.2016.BAE385, PMID: 27188468

[21] Liu Y Gao J Peng M Meng H Ma H Cai P. A review on central nervous system effects of Gastrodin. Front Pharmacol. (2018) 9:24. doi: 10.3389/fphar.2018.00024, PMID: 29456504 PMC5801292

[22] Hu F Xu L Liu ZH Ge MM Ruan DY Wang HL. Developmental lead exposure alters synaptogenesis through inhibiting canonical Wnt pathway *in vivo* and *in vitro*. PLoS ONE. (2014) 9:e101894. doi: 10.1371/journal.pone.0101894, PMID: 24999626 PMC4084981

[23] Liu CM Tian ZK Zhang YJ Ming QL Ma JQ Ji LP. Effects of Gastrodin against Lead-induced brain injury in mice associated with the Wnt/Nrf 2 pathway. Nutrients. (2020) 12:1805. doi: 10.3390/nu12061805, PMID: 32560430 PMC7353406

[24] Yong W Xing TR Wang S Chen L Hu P Li CC. Protective effects of gastrodin on lead-induced synaptic plasticity deficits in rat hippocampus. Planta Med. (2009) 75:1112–7. doi: 10.1055/s-0029-1185452, PMID: 19291610

[25] Huang ZB Wu Z Chen FK Zou LB. The protective effects of phenolic constituents from *Gastrodia elata* on the cytotoxicity induced by KCl and glutamate. Arch Pharm Res. (2006) 29:963–8. doi: 10.1007/BF02969279, PMID: 17146964

[26] An H Kim IS Koppula S Kim BW Park PJ Lim BO. Protective effects of *Gastrodia elata* Blume on MPP+-induced cytotoxicity in human dopaminergic SH-SY5Y cells. J Ethnopharmacol. (2010) 130:290–8. doi: 10.1016/j.jep.2010.05.006, PMID: 20470875

[27] Kumar H Kim IS More SV Kim BW Bahk YY Choi DK. Gastrodin protects apoptotic dopaminergic neurons in a toxin-induced Parkinson's disease model. Evid Based Complement Alternat Med. (2013) 2013:514095. doi: 10.1155/2013/514095 23533492 PMC3603713

[28] Jiang G Hu Y Liu L Cai J Peng C Li Q. Gastrodin protects against MPP(+)-induced oxidative stress by up regulates heme oxygenase-1 expression through p 38 MAPK/Nrf2 pathway in human dopaminergic cells. Neurochem Int. (2014) 75:79–88. doi: 10.1016/j.neuint.2014.06.003 24932697

[29] Gay NH Phopin K Suwanjang W Songtawee N Ruankham W Wongchitrat P. Neuroprotective effects of phenolic and carboxylic acids on oxidative stress-induced toxicity in human neuroblastoma SH-SY5Y cells. Neurochem Res. (2018) 43:619–36. doi: 10.1007/s11064-017-2463-x, PMID: 29417471

[30] Baral S Pariyar R Yoon CS Kim DC Yun JM Jang SO. Effects of Gastrodiae rhizoma on proliferation and differentiation of human embryonic neural stem cells. Asian Pac J Trop Med. (2015) 8:792–7. doi: 10.1016/j.apjtm.2015.09.004, PMID: 26522293

[31] Ramachandran U Manavalan A Sundaramurthi H Sze SK Feng ZW Hu JM. Tianma modulates proteins with various neuro-regenerative modalities in differentiated human neuronal SH-SY5Y cells. Neurochem Int. (2012) 60:827–36. doi: 10.1016/j.neuint.2012.03.012, PMID: 22710396

[32] Manavalan A Ramachandran U Sundaramurthi H Mishra M Sze SK Hu JM. *Gastrodia elata* Blume (tianma) mobilizes neuro-protective capacities. Int J Biochem Mol Biol. (2012) 3:219–41. PMID: 22773961 PMC3388733

[33] Wu S Huang R Zhang R Xiao C Wang L Luo M. Gastrodin and Gastrodigenin improve energy metabolism disorders and mitochondrial dysfunction to antagonize vascular dementia. Molecules (Basel, Switzerland). (2023) 28:2598. doi: 10.3390/molecules28062598, PMID: 36985572 PMC10059574

[34] Sundaramurthi H Manavalan A Ramachandran U Hu JM Sze SK Heese K. Phenotyping of tianma-stimulated differentiated rat neuronal b104 cells by quantitative proteomics. Neurosignals. (2012) 20:48–60. doi: 10.1159/000331492, PMID: 22094351

[35] Li X Fan R Xiang J Yuan Y Mao X Zhou N. P-hydroxy benzaldehyde facilitates reprogramming of reactive astrocytes into neurons via endogenous transcriptional regulation. Int J Neurosci. (2023) 133:1096–108. doi: 10.1080/00207454.2022.2049775 35321633

[36] Lin YE Lin CH Ho EP Ke YC Petridi S Elliott CJ. Glial Nrf2 signaling mediates the neuroprotection exerted by *Gastrodia elata* Blume in Lrrk2-G2019S Parkinson's disease. eLife. (2021) 10:e73753. doi: 10.7554/eLife.73753 34779396 PMC8660019

[37] Lu C Qu S Zhong Z Luo H Lei SS Zhong HJ. The effects of bioactive components from the rhizome of *gastrodia elata* blume (Tianma) on the characteristics of Parkinson's disease. Front Pharmacol. (2022) 13:963327. doi: 10.3389/fphar.2022.963327, PMID: 36532787 PMC9748092

[38] Meng F Guo Z Hu Y Mai W Zhang Z Zhang B. CD73-derived adenosine controls inflammation and neurodegeneration by modulating dopamine signalling. Brain J Neurol. (2019) 142:700–18. doi: 10.1093/brain/awy351, PMID: 30689733

[39] Chen S Chen H Du Q Shen J. Targeting myeloperoxidase (MPO) mediated oxidative stress and inflammation for reducing brain ischemia injury: potential application of natural compounds. Front Physiol. (2020) 11:433. doi: 10.3389/fphys.2020.00433, PMID: 32508671 PMC7248223

[40] Huang JY Yuan YH Yan JQ Wang YN Chu SF Zhu CG. 20C, a bibenzyl compound isolated from *Gastrodia elata*, protects PC12 cells against rotenone-induced apoptosis via activation of the Nrf2/ARE/HO-1 signaling pathway. Acta Pharmacol Sin. (2016) 37:731–40. doi: 10.1038/aps.2015.154, PMID: 27180985 PMC4954759

[41] Kim IS Choi DK Jung HJ. Neuroprotective effects of vanillyl alcohol in *Gastrodia elata* Blume through suppression of oxidative stress and anti-apoptotic activity in toxin-induced dopaminergic MN9D cells. Molecules (Basel, Switzerland). (2011) 16:5349–61. doi: 10.3390/molecules16075349, PMID: 21705974 PMC6264347

[42] Zhao M Zhou Y Sheng R Zhang H Xiang J Wang J. Gastrodin relieves Parkinson's disease-related motor deficits by facilitating the MEK-dependent VMAT2 to maintain dopamine homeostasis. Phytomedicine. (2024) 132:155819. doi: 10.1016/j.phymed.2024.155819 38885579

[43] Sharma N Khurana N Muthuraman A Utreja P. Pharmacological evaluation of vanillic acid in rotenone-induced Parkinson's disease rat model. Eur J Pharmacol. (2021) 903:174112. doi: 10.1016/j.ejphar.2021.174112, PMID: 33901458

[44] Yang TT Zhou HJ Zeng CY Chen C Du JR. Protective effect of novel gastrodin derivatives on Alzheimer’s disease model mice. Chinese J Mod Appl Pharm. (2019) 36:537–41. doi: 10.13748/j.cnki.issn1007-7693.2019.05.005

[45] Zhang TH Huang CM Gao X Wang JW Hao LL Ji Q. Gastrodin inhibits high glucose-induced human retinal endothelial cell apoptosis by regulating the SIRT1/TLR4/NF-κBp65 signaling pathway. Mol Med Rep. (2018) 17:7774–80. doi: 10.3892/mmr.2018.8841, PMID: 29620267

[46] Lin ZC Wen GQ Lv Y Zheng GQ Ou YF. Study on neuroprotective effect of gastrodin on rats with Alzheimer’s disease. J Guangxi Med Univ. (2020) 37:1435–41. doi: 10.16190/j.cnki.45-1211/r.2020.08.007

[47] Abuthawabeh R Abuirmeileh AN Alzoubi KH. The beneficial effect of vanillin on 6-hydroxydopamine rat model of Parkinson's disease. Restor Neurol Neurosci. (2020) 38:369–73. doi: 10.3233/RNN-201028, PMID: 32986633

[48] Doo AR Kim SN Hahm DH Yoo HH Park JY Lee H. *Gastrodia elata* Blume alleviates L-DOPA-induced dyskinesia by normalizing FosB and ERK activation in a 6-OHDA-lesioned Parkinson's disease mouse model. BMC Complement Altern Med. (2014) 14:107. doi: 10.1186/1472-6882-14-107 24650244 PMC3994477

[49] Yan J Yang Z Zhao N Li Z Cao X. Gastrodin protects dopaminergic neurons via insulin-like pathway in a Parkinson's disease model. BMC Neurosci. (2019) 20:31. doi: 10.1186/s12868-019-0512-x, PMID: 31208386 PMC6580469

[50] Kim HJ Moon KD Lee DS Lee SH. Ethyl ether fraction of *Gastrodia elata* Blume protects amyloid beta peptide-induced cell death. J Ethnopharmacol. (2003) 84:95–8. doi: 10.1016/S0378-8741(02)00290-8, PMID: 12499082

[51] Ng CF Ko CH Koon CM Xian JW Leung PC Fung KP. The aqueous extract of rhizome of *Gastrodia elata* protected Drosophila and PC12 cells against Beta-amyloid-induced neurotoxicity. Evid Based Complement Alternat Med. (2013) 2013:516741. doi: 10.1155/2013/516741 24174977 PMC3794658

[52] Yu X Tao J Xiao T Duan X. 4, 4′-methylenediphenol reduces Aβ-induced toxicity in a *Caenorhabditis elegans* model of Alzheimer's disease. Front Aging Neurosci. (2024) 16:1393721. doi: 10.3389/fnagi.2024.1393721, PMID: 38872629 PMC11171718

[53] Zhou NN Zhu R Zhao XM Liang P. Zhonghua bing li xue za zhi =. Chinese J Pathol. (2016) 45:780–5. doi: 10.3760/cma.j.issn.0529-5807.2016.11.007, PMID: 27821233

[54] Zhang JS Zhou SF Wang Q Guo JN Liang HM Deng JB. Gastrodin suppresses BACE1 expression under oxidative stress condition via inhibition of the PKR/eIF2α pathway in Alzheimer's disease. Neuroscience. (2016) 325:1–9. doi: 10.1016/j.neuroscience.2016.03.024 26987953

[55] Shi X Luo Y Yang L Duan X. Protective effect of *Gastrodia elata* Blume in a *Caenorhabditis elegans* model of Alzheimer's disease based on network pharmacology. Biomed Rep. (2023) 18:37. doi: 10.3892/br.2023.1620, PMID: 37113386 PMC10126622

[56] Hu Y Li C Shen W. Gastrodin alleviates memory deficits and reduces neuropathology in a mouse model of Alzheimer's disease. Neuropathology: official journal of the Japanese society of. Neuropathology. (2014) 34:370–7. doi: 10.1111/neup.12115, PMID: 24661139

[57] Huang GB Zhao T Muna SS Jin HM Park JI Jo KS. Therapeutic potential of *Gastrodia elata* Blume for the treatment of Alzheimer's disease. Neural Regen Res. (2013) 8:1061–70. doi: 10.3969/j.issn.1673-5374.2013.12.001, PMID: 25206400 PMC4145891

[58] Shi R Zheng CB Wang H Rao Q Du T Bai C. Gastrodin alleviates vascular dementia in a 2-VO-vascular dementia rat model by altering amyloid and tau levels. Pharmacology. (2020) 105:386–96. doi: 10.1159/000504056, PMID: 31752010

[59] Liu B Gao JM Li F Gong QH Shi JS. Gastrodin attenuates bilateral common carotid artery occlusion-induced cognitive deficits via regulating Aβ-related proteins and reducing autophagy and apoptosis in rats. Front Pharmacol. (2018) 9:405. doi: 10.3389/fphar.2018.00405, PMID: 29755351 PMC5932202

[60] Ding Y Bao X Lao L Ling Y Wang Q Xu S. P-Hydroxybenzyl alcohol prevents memory deficits by increasing neurotrophic factors and decreasing inflammatory factors in a mice model of Alzheimer's disease. J Alzheimer's Dis. (2019) 67:1007–19. doi: 10.3233/JAD-180910, PMID: 30776009

[61] Ojemann LM Nelson WL Shin DS Rowe AO Buchanan RA. Tian ma, an ancient Chinese herb, offers new options for the treatment of epilepsy and other conditions. Epilepsy Behav. (2006) 8:376–83. doi: 10.1016/j.yebeh.2005.12.009, PMID: 16461011

[62] Mori A Yokoi I Noda Y Willmore LJ. Natural antioxidants may prevent posttraumatic epilepsy: a proposal based on experimental animal studies. Acta Med Okayama. (2004) 58:111–8. doi: 10.18926/AMO/32111, PMID: 15471432

[63] An SJ Park SK Hwang IK Choi SY Kim SK Kwon OS. Gastrodin decreases immunoreactivities of gamma-aminobutyric acid shunt enzymes in the hippocampus of seizure-sensitive gerbils. J Neurosci Res. (2003) 71:534–43. doi: 10.1002/jnr.10502, PMID: 12548709

[64] Shin EJ Bach JH Nguyen TT Jung BD Oh KW Kim MJ. *Gastrodia Elata* Bl attenuates cocaine-induced conditioned place preference and convulsion, but not behavioral sensitization in mice: importance of GABA (a) receptors. Curr Neuropharmacol. (2011) 9:26–9. doi: 10.2174/157015911795017326, PMID: 21886556 PMC3137195

[65] Ha JH Lee DU Lee JT Kim JS Yong CS Kim JA. 4-Hydroxybenzaldehyde from *Gastrodia elata* B1. Is active in the antioxidation and GABAergic neuromodulation of the rat brain. J Ethnopharmacol. (2000) 73:329–33. doi: 10.1016/S0378-8741(00)00313-5, PMID: 11025174

[66] Yang CS Chiu SC Liu PY Wu SN Lai MC Huang CW. Gastrodin alleviates seizure severity and neuronal excitotoxicities in the rat lithium-pilocarpine model of temporal lobe epilepsy via enhancing GABAergic transmission. J Ethnopharmacol. (2021) 269:113751. doi: 10.1016/j.jep.2020.113751, PMID: 33359863

[67] Chen L Liu X Wang H Qu M. Gastrodin attenuates Pentylenetetrazole-induced seizures by modulating the mitogen-activated protein kinase-associated inflammatory responses in mice. Neurosci Bull. (2017) 33:264–72. doi: 10.1007/s12264-016-0084-z, PMID: 27909971 PMC5567506

[68] Kim HJ Moon KD Oh SY Kim SP Lee SR. Ether fraction of methanol extracts of *Gastrodia elata*, a traditional medicinal herb, protects against kainic acid-induced neuronal damage in the mouse hippocampus. Neurosci Lett. (2001) 314:65–8. doi: 10.1016/s0304-3940(01)02296-0, PMID: 11698148

[69] Hsieh CL Chiang SY Cheng KS Lin YH Tang NY Lee CJ. Anticonvulsive and free radical scavenging activities of *Gastrodia elata* Bl. In kainic acid-treated rats. Am J Chin Med. (2001) 29:331–41. doi: 10.1142/S0192415X01000356, PMID: 11527075

[70] Hsieh CL Lin JJ Chiang SY Su SY Tang NY Lin GG. *Gastrodia elata* modulated activator protein 1 via c-Jun N-terminal kinase signaling pathway in kainic acid-induced epilepsy in rats. J Ethnopharmacol. (2007) 109:241–7. doi: 10.1016/j.jep.2006.07.024, PMID: 16934418

[71] Hsieh CL Chang CH Chiang SY Li TC Tang NY Pon CZ. Anticonvulsive and free radical scavenging activities of vanillyl alcohol in ferric chloride-induced epileptic seizures in Sprague-Dawley rats. Life Sci. (2000) 67:1185–95. 10954052 10.1016/s0024-3205(00)00706-2

[72] Shao H Yang Y Qi AP Hong P Zhu GX Cao XY. Gastrodin reduces the severity of status epilepticus in the rat pilocarpine model of temporal lobe epilepsy by inhibiting Nav 1.6 sodium currents. Neurochem Res. (2017) 42:360–74. doi: 10.1007/s11064-016-2079-6, PMID: 27743286

[73] Zhou BH Li XJ Liu M Wu Z Hu XM. Antidepressant-like activity of the *Gastrodia elata* ethanol extract in mice. Fitoterapia. (2006) 77:592–4. doi: 10.1016/j.fitote.2006.06.016, PMID: 17052862

[74] Chen PJ Hsieh CL Su KP Hou YC Chiang HM Lin IH. The antidepressant effect of *Gastrodia elata* Bl. On the forced-swimming test in rats. The American journal of. Chin Med. (2008) 36:95–106. doi: 10.1142/S0192415X08005618, PMID: 18306453

[75] Chen PJ Hsieh CL Su KP Hou YC Chiang HM Sheen LY. Rhizomes of *Gastrodia elata* B (L) possess antidepressant-like effect via monoamine modulation in subchronic animal model. Am J Chin Med. (2009) 37:1113–24. doi: 10.1142/S0192415X09007533 19938220

[76] Lin YE Lin SH Chen WC Ho CT Lai YS Panyod S. Antidepressant-like effects of water extract of *Gastrodia elata* Blume in rats exposed to unpredictable chronic mild stress via modulation of monoamine regulatory pathways. J Ethnopharmacol. (2016) 187:57–65. doi: 10.1016/j.jep.2016.04.032, PMID: 27109341

[77] Lin SH Chen WC Lu KH Chen PJ Hsieh SC Pan TM. Down-regulation of slit-Robo pathway mediating neuronal cytoskeletal remodeling processes facilitates the antidepressive-like activity of *Gastrodia elata* Blume. J Agric Food Chem. (2014) 62:10493–503. doi: 10.1021/jf503132c, PMID: 25197951

[78] Chen WC Lai YS Lin SH Lu KH Lin YE Panyod S. Anti-depressant effects of *Gastrodia elata* Blume and its compounds gastrodin and 4-hydroxybenzyl alcohol, via the monoaminergic system and neuronal cytoskeletal remodeling. J Ethnopharmacol. (2016) 182:190–9. doi: 10.1016/j.jep.2016.02.001, PMID: 26899441

[79] Wang R Ren Q Gao D Paudel YN Li X Wang L. Ameliorative effect of *Gastrodia elata* Blume extracts on depression in zebrafish and cellular models through modulating reticulon 4 receptors and apoptosis. J Ethnopharmacol. (2022) 289:115018. doi: 10.1016/j.jep.2022.115018, PMID: 35092824

[80] Jiang N Yao C Zhang Y Chen Y Chen F Luo Y. Antidepressant effects of Parishin C in chronic social defeat stress-induced depressive mice. J Ethnopharmacol. (2024) 325:117891. doi: 10.1016/j.jep.2024.117891, PMID: 38331122

[81] Lin YE Chou ST Lin SH Lu KH Panyod S Lai YS. Antidepressant-like effects of water extract of *Gastrodia elata* Blume on neurotrophic regulation in a chronic social defeat stress model. J Ethnopharmacol. (2018) 215:132–9. doi: 10.1016/j.jep.2017.12.044, PMID: 29288827

[82] Wu YN Wen SH Zhang W Yu SS Yang K Liu D. *Gastrodia elata* BI.: a comprehensive review of its traditional use, botany, phytochemistry, pharmacology, and pharmacokinetics. Evid Based Complement Alternat Med. (2023) 2023:5606021. doi: 10.1155/2023/5606021, PMID: 37114145 PMC10129437

[83] Shin EJ Kim JM Nguyen XK Nguyen TT Lee SY Jung JH. Effects of *gastrodia elata* bl on phencyclidine-induced schizophrenia-like psychosis in mice. Curr Neuropharmacol. (2011) 9:247–50. doi: 10.2174/157015911795017263, PMID: 21886599 PMC3137192

[84] Shin EJ Whang WK Kim S Bach JH Kim JM Nguyen XK. Parishin C attenuates phencyclidine-induced schizophrenia-like psychosis in mice: involvements of 5-HT1A receptor. J Pharmacol Sci. (2010) 113:404–8. doi: 10.1254/jphs.10040SC, PMID: 20644336

[85] Hsieh MT Peng WH Wu CR Wang WH. The ameliorating effects of the cognitive-enhancing Chinese herbs on scopolamine-induced amnesia in rats. Phytother Res. (2000) 14:375–7. PMID: 10925408 10.1002/1099-1573(200008)14:5<375::aid-ptr593>3.0.co;2-5

[86] Wu CR Hsieh MT Huang SC Peng WH Chang YS Chen CF. Effects of *Gastrodia elata* and its active constituents on scopolamine-induced amnesia in rats. Planta Med. (1996) 62:317–21. doi: 10.1055/s-2006-957892, PMID: 8792662

[87] Chen PJ Liang KC Lin HC Hsieh CL Su KP Hung MC. *Gastrodia elata* Bl. Attenuated learning deficits induced by forced-swimming stress in the inhibitory avoidance task and Morris water maze. J Med Food. (2011) 14:610–7. doi: 10.1089/jmf.2010.1209, PMID: 21554135

[88] Hsieh MT Wu CR Chen CF. Gastrodin and p-hydroxybenzyl alcohol facilitate memory consolidation and retrieval, but not acquisition, on the passive avoidance task in rats. J Ethnopharmacol. (1997) 56:45–54. doi: 10.1016/S0378-8741(96)01501-2, PMID: 9147253

[89] Lee HE Lee YW Park SJ Jeon SJ Kim E Lee S. 4-Hydroxybenzyl methyl ether improves learning and memory in mice via the activation of dopamine D1 receptor signaling. Neurobiol Learn Mem. (2015) 121:30–8. doi: 10.1016/j.nlm.2015.03.004, PMID: 25843525

[90] Wang X Yan S Wang A Li Y Zhang F. Gastrodin ameliorates memory deficits in 3, 3′-iminodipropionitrile-induced rats: possible involvement of dopaminergic system. Neurochem Res. (2014) 39:1458–66. doi: 10.1007/s11064-014-1335-x, PMID: 24842556

[91] Song Z Luo G Han C Jia G Zhang B. Potential targets and action mechanism of Gastrodin in the treatment of attention-deficit/hyperactivity disorder: bioinformatics and network pharmacology analysis. Evid Based Complement Alternat Med. (2022) 2022:3607053. doi: 10.1155/2022/3607053 36133787 PMC9484880

[92] Wu CR Hsieh MT Liao J. P-Hydroxybenzyl alcohol attenuates learning deficits in the inhibitory avoidance task: involvement of serotonergic and dopaminergic systems. Chin J Physiol. (1996) 39:265–73. PMID: 9058011

[93] Wang X Li P Liu J Jin X Li L Zhang D. Gastrodin attenuates cognitive deficits induced by 3, 3'-Iminodipropionitrile. Neurochem Res. (2016) 41:1401–9. doi: 10.1007/s11064-016-1845-9, PMID: 26869041

[94] Shuchang H Qiao N Piye N Mingwei H Xiaoshu S Feng S. Protective effects of *gastrodia elata* on aluminium-chloride-induced learning impairments and alterations of amino acid neurotransmitter release in adult rats. Restor Neurol Neurosci. (2008) 26:467–73. doi: 10.3233/RNN-2008-00431, PMID: 19096134 PMC2689815

[95] Wu LY Chen WC Tsai FS Tsai CC Wu CR Lin LW. P-Hydroxybenzyl alcohol, an active phenolic ingredient of *Gastrodia elata*, reverses the Cycloheximide-induced memory deficit by activating the adrenal gland in rats. Am J Chin Med. (2015) 43:1593–604. doi: 10.1142/S0192415X15500901, PMID: 26621444

[96] Mishra M Huang J Lee YY Chua DS Lin X Hu JM. *Gastrodia elata* modulates amyloid precursor protein cleavage and cognitive functions in mice. Biosci Trends. (2011) 5:129–38. doi: 10.5582/bst.2011.v5.3.129, PMID: 21788698

[97] Duan X Wang W Liu X Yan H Dai R Lin Q. Neuroprotective effect of ethyl acetate extract from *gastrodia elata* against transient focal cerebral ischemia in rats induced by middle cerebral artery occlusion. J Trad Chinese Med. (2015) 35:671–8. doi: 10.1016/s0254-6272(15)30158-8, PMID: 26742313

[98] Wang S Nan Y Zhu W Yang T Tong Y Fan Y. Gastrodin improves the neurological score in MCAO rats by inhibiting inflammation and apoptosis, promoting revascularization. Int J Clin Exp Pathol. (2018) 11:5343–50. PMID: 31949615 PMC6963028

[99] Kim HJ Lee SR Moon KD. Ether fraction of methanol extracts of *Gastrodia elata*, medicinal herb protects against neuronal cell damage after transient global ischemia in gerbils. Phytother Res. (2003) 17:909–12. doi: 10.1002/ptr.1246, PMID: 13680822

[100] Ng CF Ko CH Koon CM Chin WC Kwong HC Lo AW. The aqueous extract of rhizome of *Gastrodia elata* Blume attenuates locomotor defect and inflammation after traumatic brain injury in rats. J Ethnopharmacol. (2016) 185:87–95. doi: 10.1016/j.jep.2016.03.018 26979339

[101] Luo L Kim SW Lee HK Kim ID Lee H Lee JK. Gastrodin exerts robust neuroprotection in the postischemic brain via its protective effect against Zn2+−toxicity and its anti-oxidative effects in astrocytes. Anim Cells Syst. (2018) 22:429–37. doi: 10.1080/19768354.2018.1549099, PMID: 30533266 PMC6282451

[102] Shi A Xiang J He F Zhu Y Zhu G Lin Y. The phenolic components of *Gastrodia elata* improve prognosis in rats after cerebral ischemia/reperfusion by enhancing the endogenous antioxidant mechanisms. Oxidative Med Cell Longev. (2018) 2018:7642158. doi: 10.1155/2018/7642158, PMID: 29765502 PMC5885496

[103] Zeng X Zhang Y Zhang S Zheng X. A microdialysis study of effects of gastrodin on neurochemical changes in the ischemic/reperfused rat cerebral hippocampus. Biol Pharm Bull. (2007) 30:801–4. doi: 10.1248/bpb.30.801, PMID: 17409525

[104] Zeng X Zhang S Zhang L Zhang K Zheng X. A study of the neuroprotective effect of the phenolic glucoside gastrodin during cerebral ischemia *in vivo* and *in vitro*. Planta Med. (2006) 72:1359–65. doi: 10.1055/s-2006-951709, PMID: 17089323

[105] Wang D Wang Q Chen R Yang S Li Z Feng Y. Exploring the effects of *Gastrodia elata* Blume on the treatment of cerebral ischemia-reperfusion injury using UPLC-Q/TOF-MS-based plasma metabolomics. Food Funct. (2019) 10:7204–15. doi: 10.1039/c9fo01729a, PMID: 31609374

[106] Song C Fang S Lv G Mei X. Gastrodin promotes the secretion of brain-derived neurotrophic factor in the injured spinal cord. Neural Regen Res. (2013) 8:1383–9. doi: 10.3969/j.issn.1673-5374.2013.15.005, PMID: 25206433 PMC4107770

[107] Li X Xiang B Shen T Xiao C Dai R He F. Anti-neuroinflammatory effect of 3, 4-dihydroxybenzaldehyde in ischemic stroke. Int Immunopharmacol. (2020) 82:106353. doi: 10.1016/j.intimp.2020.106353 32143007

[108] Kam KY Yu SJ Jeong N Hong JH Jalin AM Lee S. P-Hydroxybenzyl alcohol prevents brain injury and behavioral impairment by activating Nrf2, PDI, and neurotrophic factor genes in a rat model of brain ischemia. Mol Cells. (2011) 31:209–15. doi: 10.1007/s10059-011-0028-4 21347705 PMC3932695

[109] Yu X Luo Y Yang L Duan X. Plasma metabonomic study on the effect of Para-hydroxybenzaldehyde intervention in a rat model of transient focal cerebral ischemia. Mol Med Rep. (2023) 28:224. doi: 10.3892/mmr.2023.13111, PMID: 37800608 PMC10577806

[110] Yu SJ Kim JR Lee CK Han JE Lee JH Kim HS. *Gastrodia elata* blume and an active component, p-hydroxybenzyl alcohol reduce focal ischemic brain injury through antioxidant related gene expressions. Biol Pharm Bull. (2005) 28:1016–20. doi: 10.1248/bpb.28.1016, PMID: 15930737

[111] Descamps E Petrault-Laprais M Maurois P Pages N Bac P Bordet R. Experimental stroke protection induced by 4-hydroxybenzyl alcohol is cancelled by bacitracin. Neurosci Res. (2009) 64:137–42. doi: 10.1016/j.neures.2009.02.005, PMID: 19428693

[112] Yu SS Zhao J Zheng WP Zhao Y. Neuroprotective effect of 4-hydroxybenzyl alcohol against transient focal cerebral ischemia via anti-apoptosis in rats. Brain Res. (2010) 1308:167–75. doi: 10.1016/j.brainres.2009.10.037, PMID: 19857470

[113] Luo L Kim SW Lee HK Kim ID Lee H Lee JK. Anti-Zn2+−toxicity of 4-Hydroxybenzyl alcohol in astrocytes and neurons contribute to a robust neuroprotective effects in the Postischemic brain. Cell Mol Neurobiol. (2018) 38:615–26. doi: 10.1007/s10571-017-0508-y, PMID: 28608001 PMC11481900

[114] Cao L Zhang D Chen J Qin YY Sheng R Feng X. G6PD plays a neuroprotective role in brain ischemia through promoting pentose phosphate pathway. Free Radic Biol Med. (2017) 112:433–44. doi: 10.1016/j.freeradbiomed.2017.08.011, PMID: 28823591

[115] Tu D Gao Y Yang R Guan T Hong JS Gao HM. The pentose phosphate pathway regulates chronic neuroinflammation and dopaminergic neurodegeneration. J Neuroinflammation. (2019) 16:255. doi: 10.1186/s12974-019-1659-1, PMID: 31805953 PMC6896486

[116] Yao YY Bian LG Yang P Sui Y Li R Chen YL. Gastrodin attenuates proliferation and inflammatory responses in activated microglia through Wnt/β-catenin signaling pathway. Brain Res. (2019) 1717:190–203. doi: 10.1016/j.brainres.2019.04.025, PMID: 31026457

[117] Qiu CW Liu ZY Zhang FL Zhang L Li F Liu SY. Post-stroke gastrodin treatment ameliorates ischemic injury and increases neurogenesis and restores the Wnt/β-catenin signaling in focal cerebral ischemia in mice. Brain Res. (2019) 1712:7–15. doi: 10.1016/j.brainres.2019.01.043, PMID: 30716287

[118] Xiao H Jiang Q Qiu H Wu K Ma X Yang J. Gastrodin promotes hippocampal neurogenesis via PDE9-cGMP-PKG pathway in mice following cerebral ischemia. Neurochem Int. (2021) 150:105171. doi: 10.1016/j.neuint.2021.105171, PMID: 34419525

[119] Chen PJ Sheen LY. Gastrodiae Rhizoma (tiān má): a review of biological activity and antidepressant mechanisms. J Tradit Complement Med. (2011) 1:31–40. doi: 10.1016/s2225-4110(16)30054-2, PMID: 24716103 PMC3942998

[120] Liu W Wang L Yu J Asare PF Zhao YQ. Gastrodin reduces blood pressure by intervening with RAAS and PPARγ in SHRs. Evid Based Complement Alternat Med. (2015) 2015:828427. doi: 10.1155/2015/828427 26587048 PMC4637485

[121] Qiu F Liu TT Qu ZW Qiu CY Yang Z Hu WP. Gastrodin inhibits the activity of acid-sensing ion channels in rat primary sensory neurons. Eur J Pharmacol. (2014) 731:50–7. doi: 10.1016/j.ejphar.2014.02.044, PMID: 24642360

[122] Huang Q Shi J Gao B Zhang HY Fan J Li XJ. Gastrodin: an ancient Chinese herbal medicine as a source for anti-osteoporosis agents via reducing reactive oxygen species. Bone. (2015) 73:132–44. doi: 10.1016/j.bone.2014.12.059, PMID: 25554600

[123] Xu X Lu Y Bie X. Protective effects of gastrodin on hypoxia-induced toxicity in primary cultures of rat cortical neurons. Planta Med. (2007) 73:650–4. doi: 10.1055/s-2007-981523, PMID: 17583824

